# Semantic guidance network for video captioning

**DOI:** 10.1038/s41598-023-43010-3

**Published:** 2023-09-26

**Authors:** Lan Guo, Hong Zhao, ZhiWen Chen, ZeYu Han

**Affiliations:** 1 School of Computer and Communication, Lanzhou University of Technology, LanZhou, 730050 China; 2 Network and Information Center, Lanzhou University of Technology, LanZhou, 730050 China

**Keywords:** Machine learning, Computer science, Electrical and electronic engineering

## Abstract

video captioning is a more challenging task that aims to generate abundant natural language descriptions, and it has become a promising direction for artificial intelligence. However, most existing methods are prone to ignore the problems of visual information redundancy and scene information omission due to the limitation of the sampling strategies. To address this problem, a semantic guidance network for video captioning is proposed. More specifically, a novel scene frame sampling strategy is first proposed to select key scene frames. Then, the vision transformer encoder is applied to learn visual and semantic information with a global view, alleviating information loss of modeling long-range dependencies caused in the encoder’s hidden layer. Finally, a non-parametric metric learning module is introduced to calculate the similarity value between the ground truth and the prediction result, and the model is optimized in an end-to-end manner. Experiments on the benchmark MSR-VTT and MSVD datasets show that the proposed method can effectively improve the description accuracy and generalization ability.

## Introduction

Video captioning aims to describe every scene given the video using natural language, and it is one of the most challenging tasks in computer vision because it requires the association of the video to text. It has been widely used in video searching^1–3^, robot video question-and-answer^4,5^ and many others. For example, automatic video captioning greatly helps users quickly screen out target content on video platforms. With the growth of neural machine translation (NMT)^6^, existing video caption methods are based on an encoder-decoder framework with recursive neural network (RNN)^7^ and convolutional neural network (CNN)^8^. The methods mainly focus on two parts of the video subtitle architecture, namely video encoder and subtitle generation. For the video encoder, the CNN-based encoder acquires a set of consecutive or equally spaced sample frames of the video and generates visual representation as input vectors for the model decoder. In particular, attention mechanism^9^ and time object diagram^10^ are also used to enhance the representativeness of the video in the video encoder. The other one is caption generation, LSTM network is adopted as decoder^11^, and complex language rules are further explored to improve the generation ability for the model. The visual encoder features and previously predicted output words are used as the input for the new module by the RNN-based decoder and gradually predicts the next word. Finally, a complete video captioning statement is generated.

Different from image captioning merely needing to comprehend the static content of the single image and generate the description, video captioning can understand the context information of the video in time sequence, rather than focusing on a single frame. Here, we consider that a high similarity exists between adjacent frames in the video, continuous video frames usually do not provide independent information. Therefore, each frame is regarded as a separate information unit, which may be not be the best choice for understanding video content. As mentioned in literature^12^, compared with natural language, it is more reasonable to divide video into scene-based information units to understand the information expressed in the video. Therefore, we use scenario-based information grouping to understand video. For example, a video is composed of five scenes of information. In each scene, the largest difference frames are obtained, and finally, the input frame set of visual feature extractors is formed.

The contribution of this paper can be summarized as follows:

1. We propose a key scene frame screening method based on multi-scale image similarity measurement. By comparing the similarity of each frame, the best keyframe expression of the video in each scene is learned.
2. We introduce an image captioning model to learn the description of the keyframe, and word2vec is used to transform the generated image captions vector into video semantic features. Capitalizes on the image captioning model’s proficiency in generating informative and contextually relevant descriptions for static images, thereby extending its utility to the dynamic realm of video content.
3. Encoder blocks of Vision Transformer are used as feature encoder of the proposed model to achieve long-distance modeling and the feature hard encoder of CNN is learned from a global perspective to reduce loss of the hidden layer information in the middle of the encoder.
4. A non-parametric metric learning module is proposed to optimize model parameters using the ground truth and the prediction result, and the description ability of our model is further enhanced to dynamically adapt video semantic information.

## Related work

Video captioning is a complex and challenging task as it contains two different modalities, CV (Computer Vision) and NLP (Natural Language Processing). In this section, the relevant works to imaging and video captioning are summarized respectively.

### Image caption

Image captioning^13–15^ is a fundamental task in vision-language understanding. Previous works mainly focus on the template-based method^16,17^ which needs to set multiple filling templates in advance. The filling template forms an empty slot by object relationship and attribute tag, generating image captioning statements. The generative process has some obvious limitations. For example, the generated sentences are fixed due to the limitation of limited templates designed by hand, and the generated sentences do not perform well in terms of naturalness and diversity.

Recently, the framework of the encoder-decoder has been widely used in image captioning based on deep learning. Specifically, CNNs are often used to encode image features. RNNs are used frequently in the extraction of image features and in the generation of text descriptions phase. In recent years, research on graphical multimodality has made great progress. For example, Hong et al.^18^ combines the traditional image feature extraction techniques with the bowed word packet model. They improve the representation of semantic information of images by using TF-IDF in NLP to weight words. Vinyals et al.^19^ has proposed a neural image caption (NIC) model. They introduced a novel approach to enhance the long-term memory capability of the model, which is to replace the RNN in the m-RNN model using a long short-term memory (LSTM) network. The approach significantly improves the generation of image captions. The above work uses an encoder-decoder structure. First, the image semantic feature information is extracted by CNN. Then, the image caption statements are generated after decoding by RNN. Due to the feature extraction ability and natural language generation ability of the deep neural network, the generated description sentence structure is reasonable, smooth, and natural. Later on, Fan et al.^20^ proposes a novel grounded attention feedback model for automatic sentence generation from images. The generated text is used to rectify interest regions of images. In this strategy, the limitation of the one-way attention mechanism is broken out. The image caption generation method based on deep learning does not depend on the template with a single language. It is vivid and easy to comprehend but also has better training generation effects and speed than the template method. Li et al.^21^ introduced a framework that integrates attribute-based representations and attention mechanisms to enhance the understanding of visual content and generate descriptive language. By incorporating attributes, this method aims to capture finer-grained details in images and improve the quality of generated language descriptions. This work advances the synergy between the fields of vision and language by exploring the interplay between attributes and attention mechanisms. On the other hand, Ning et al.^22^ proposed an approach that focuses on learning representations and maintaining semantic consistency between remote sensing images and voice descriptions. By ensuring that the learned representations capture meaningful and semantically coherent information, improved retrieval performance is expected. This work emphasizes the significance of semantic coherence in learned representations, contributing to the fields of remote sensing and cross-modal retrieval.

### Video caption

The video captioning task is spatiotemporal dynamic, multi-modal, and content diverse. So much so that it is more complex compared to image captioning tasks. In the early stage, the work of video subtitle adopts the time-based method^23,24^ to define a sentence template with grammatical rules. The sentence is parsed into subject, verb, and object, each of which is consistent with the video content. If a template with a fixed syntactic structure is predefined, the generated sentence will be fixed and single. In recent years, CNNs and RNNs have been commendably applied to various tasks. As a result, sequence learning methods^25–27^ are extensively used to caption video tasks with flexible syntactic structures. In recent years, deep learning models including CV, multimedia information retrieval, video understanding, and other tasks have achieved many excellent results. There are some typical examples such as action recognition^28^, and video question answering systems^29^. Therefore, the index results of the video captioning method based on deep learning are also significantly improved. The encoder is utilized to learn the visual coding representation of video. The decoder structure of generating sentences is used to convert the learned video coding representation into natural language description text with a more flexible structure and more human understanding. Venugopalan et al.^7^ used the sequence-to-sequence model. The LSTM is used to encode visual information. And then the corresponding sentences are generated through the modified LSTM. Xu et al.^30^ design a 3D-CNN encoder, which is used to generate visual features from video time and regions. Nian et al.^31^ presents an innovative approach to video captioning that focuses on learning explicit video attributes from mid-level representations. By explicitly modeling video attributes, the method enhances the semantic richness of generated captions. The paper emphasizes the importance of capturing diverse video characteristics, such as object attributes and actions, to enrich the context of captions. Through an intricate interplay of attribute learning and caption generation, the proposed technique contributes to the enhancement of video caption quality, paving the way for more contextually relevant and informative descriptions. Chen et al.^32^ propose the TDConvED module, which makes clear the learning action according to the theme and video dynamics at the same time. Zheng et al.^33^ introduce the transformer structure in the video captioning task. This approach reduces the redundancy in the video representation. The above methods have made great progress in natural language generation, but most models need to input all video frames or sample a fixed number of video frames at equal intervals. Video is known to consist of multiple consecutive video frames containing highly repetitive information. Taking advantage of this feature, researchers have designed some methods to encode video into information units through video captioning, a practice that mimics the way humans understand video content. Tang et al.^34^ introduce a pioneering approach that leverages the CLIP (Contrastive Language-Image Pretraining) framework for video captioning tasks. By extending the capabilities of CLIP to handle video content, the authors propose a novel method for generating captions that succinctly describe the visual content within videos. The fusion of image and text understanding is harnessed to enhance the model’s comprehension of video semantics, resulting in improved caption quality. This work showcases the synergy between multimodal pretraining and video captioning, contributing to the advancement of captioning techniques by effectively incorporating a state-of-the-art image-text embedding framework. HRNE^35^ uses a hierarchical LSTM network. Specifically, the input video is first encoded into a two-level abstraction. Later, BAE^36^ makes the assumption that a video is a set of continuous events. Then, the HRNE model is improved by seeking out the video hierarchical structure. Pick net^37^ assumes that all frames sampled at equal intervals do not necessarily contain meaningful information, but only select frames with certain significance for video captioning for model training. The above method encodes the input video by merging or discarding intermediate frames. Therefore, these methods do not consider the captions being generated. The same video features are used throughout the decoding process after the input video has been encoded.

To alleviate this problem, a semantic guidance network for video captioning is proposed. First, a scene frame sampling method is proposed using the image similarity algorithm for the model to perform critical scene frame selection, and then a semantic guided learning video captioning network structure is implemented based on metric learning. Which, we measure the similarity of each frame by MS-SSIM algorithm, remove the very similar video frames and keep the frames with large differences as the visual representation information of the video. Then, 2D, as well as optical flow and 3D visual features of the scene frames, are extracted, image caption of the scene frames are performed based on a pre-trained image caption generation system on the COCO dataset, and the generated description statements are converted into vector representations as semantic information of the video by a word2vec model. Secondly, the Transformer Encoder Block is used to form a model structure encoder to learn the above visual and semantic information with a global view and to reduce the loss of information in the middle hidden layer of the encoder for modeling long-range dependencies while learning a mixed representation of local and global features in the shallow layer. Finally, the output results of the decoder and the ground truth are used for Non-parametric metric learning, and the model is optimized end-to-end by minimizing the difference between the generated description and the ground truth. Through extensive experiments on two benchmark datasets, MSR-VTT and MSVD, it is shown that the proposed model can effectively enhance the model description ability of accuracy and generalization.

## Problem formulation

Using natural language to automatically describe video content is an important task, and it has become a significant challenge in recent years. However, in the feature extraction phase, most existing models also use a random sample or uniform sample, and then the video caption content is generated via a high dimensional feature encoder and decoder. For example, literature^38^ uniformly samples 30 frames from each video, and small video clips around the sample frames are provided for feature extraction by 2D-CNN and 3D-CNN. Simultaneously, appearance and motion features are spliced to form frame representation information which is sent into the encoder-decoder constituted by LSTM to generate the final caption. Specifically, continuous video frames usually contain very similar semantic information, which can be divided into different information units when humans understand video. However, most video caption methods encode video into an information unit and after that processing, may discard or aggregate video frames with repeated information. Our purpose is to calculate the similarity between each frame of video through the MS-SSIM algorithm, to eliminate highly similar video frames. The original semantic information of the video with as few frames as possible is retained.

We define the dataset as $D_{base}$ and total *N* videos, *i.e.*, $V= \{V_1, V_2, \ldots , V_n\}$. For a given video $V_{i}$ has a total of *S* frames and *M* corresponding natural language description text *T*, where $V_{i}= \{F_{i1}, F_{i2}, \ldots , F_{is}\}$, $T_{i}= \{t_{i1}, t_{i2}, \ldots , t{_im}\}$, *i.e.*, $D_{base} = \{(V_{i}, T_{i}), 1\le i \le N\}$. Following the previous definition in literature^39^, split proportion of $D_{base}$ is set to 0.8, 0.1 and 0.1, and $D_{base\_train}$, $D_{base\_val}$ and $D_{base\_test}$ are constructed, respectively, *i.e.*, $D_{base\_train}=\{(V{_i}, T{_i}); 1\le i\le 0.8\times N\}$, $D_{base\_val}=\{(V_i, T_i), 0.8\times N< i\le 0.9\times N\}$, $D_{base\_test}=\{(V_{i}, T_{i}), 0.9\times N<i \le N\}$.

*S* frames from a video are divided by using the FFmpeg, *i.e.*, $V_{i} = \{F_{i1}, F_{i2},..., F_{is}\}$. The *X* frame with the largest difference is reserved as the adaptive sampling frame set F of each video by calculating the structural similarity between frames, *i.e.*, $F = \{ f_1, f_2, ..., f_x\}$. *X* the frame is taken as the input of 2D and 3D CNN during the encoder stage, and then $p^d$ feature vector $U^d$ of 2D-CNN and $q^d$ feature vector $Z^d$ of 3D-CNN are obtained, *i.e.*, $U^d = \{u^d_{1}, u^d_2, u^d_3,..., u^d_p\}$ and $Z^d = \{z^d_1, z^d_2, z^d_3, ..., z^d_q\}$. Next, equal interval 5 frames from the adaptive frame set *F* are selected as the input of the static image caption model on COCO datasets, and 5 corresponding text descriptions are output. Finally, the corresponding description is mapped as $R_d$ vector *W* via word2vec model, *i.e.*, $W^d = \{w^d_1, w^d_2, w^d_3, ..., w^d_r\}$. The final description sentence is represented as *Y*, *i.e.*, $Y = \{y_1, y_2,..., y_m\}$.

## Methodology

### Overview

**Figure 1:**
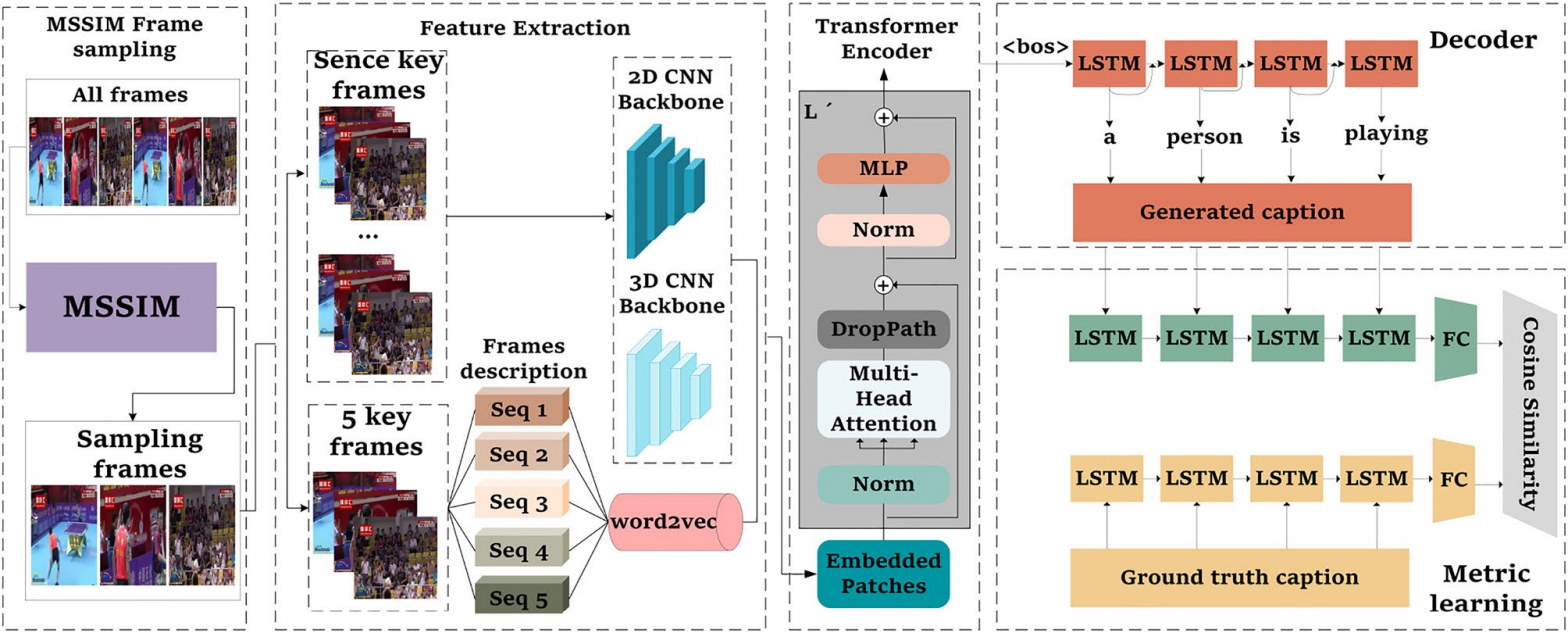
The overall overview of our method. Our model is trained in five stages. In the first stage (Frame Sampling), the frame similarity is calculated by the MS-SSIM algorithm, and the image with a large difference in semantic similarity is selected as the keyframe. In the second stage (Feature Extraction), the extraction of 2D and 3D CNN features of video and natural language captioning of images generated by keyframes. In the third stage (ViT Encoder), The encoder block of the transformer architecture is used as the feature encoder of the model structure. In the fourth stage (Decoder), The LSTM is used as the decoder of the model structure. In the fifth stage (Non-parametric Metric Learning), a reverse auxiliary learning module is designed to reinforce learned of the video caption via computing the loss Generated Caption $\rightarrow$ Ground Truth.

Most existing video captioning methods mainly use encoder-decoder neural network structures. The architecture has two parts: encoder & decoder. It has been proven to be an advanced technology of machine translation. In Fig. 1, our model is made up of four components: (a) Adaptive frame sampling module. The frame similarity is calculated by the MS-SSIM algorithm, and the image with a large difference in semantic similarity is selected as the keyframe. (b) Feature extraction module. Extraction of 2D and 3D CNN features of video and natural language captioning of images generated by keyframes. (c) Feature encoding module. The encoder block of the transformer architecture is used as the feature encoder of the model structure. It can not only complete long-range modeling but also generate video representation vectors containing contextual semantic information. (d) Decoder module. Decode the high-dimensional vector output from the encoder to generate natural language captions for the video. (e) Metrics learning module. First, the natural language captions generated by the decoder are subjected to cosine similarity measures with the real captions. Then, the model is optimized again in the training process. In addition, we validate the model performance in melting experiments.

In order to avoid the redundant information of visual features extracted by continuous frames, we adopt the MS-SSIM algorithm to realize adaptive sampling frame. It culls highly similar frames and better preserves the visual information of the video. The adaptive scene frame selector based on MS-SSIM greatly reduces redundant frames and computational costs and ensures that key information is not lost. A details explanation is given in Section “Adaptive scene frame selector”. Then, the sampled frames are sent to the feature extraction module to extract visual features as well as single-frame captions. Although the LSTM network has obvious advantages in the serialization of data, it has the problem of long-distance dependence, and its sequence-dependent structure hinders the parallelization problem among training samples. In addition, the memory limitation for long sequences also hinders the batch processing of training samples. On the other hand, the transformer structure is a model structure that avoids loops and completely relies on an attention mechanism to model the global dependence of input and output. Therefore, we adopt the Vision Transformer (ViT) encoder. First, the video feature map extracted by the backbone network is divided into multiple patches using a convolution block, and each patch is treated as a sub-patch in the sequence. Then, a linear layer is used to map the sub-patch to a visual word vector (also known as a token). These visual tokens are stitched together and converted into a visual sentence vector. Finally, the converted visual sentence vector is sent to the ViT encoder block for vector encoding to learn fine-grained features. The method used to extract and form a feature map for a data sample from the feature extraction stage and transfer it to the transformer encoder stage can be represented by the following Eq. 1:

1 $$\begin{aligned} {F_{\text{ seq } }=\left[ f_1, f_2, \ldots , f_N\right] , f_i=W_p \cdot P_i+b_p} \end{aligned}$$

where $F_{seq}$ is the sequence of feature vectors, $f_i$ is the *i*-th feature vector, *N* is the number of patches in the feature map, $P_i$ is the *i*-th patch, $W_p$ and $b_p$ are the weights and biases of the convolution block, respectively. In this method, the video feature map is first divided into patches using a convolution block. Each patch is then treated as a sub-patch in the sequence and transformed into a feature vector $f_i$ using the convolution operation. These feature vectors are concatenated to form the sequence of feature vectors $F_{seq}$, which is then fed into the transformer encoder stage, as detailed in Section “Visual and sentence encoder”. Finally, metric learning is introduced in Section “Nonparametric metric learning module”. It calculates the cosine distance between the sentence output from each decoder and the real sentence corresponding to that video by non-parametric metric learning and penalizes or rewards the model loss based on the metric value. As much as possible, real sentences are used to enhance the encoded visual features. This allows the model to be reoptimized during training and improves the model’s performance.

### Adaptive scene frame selector

**Figure 2:**
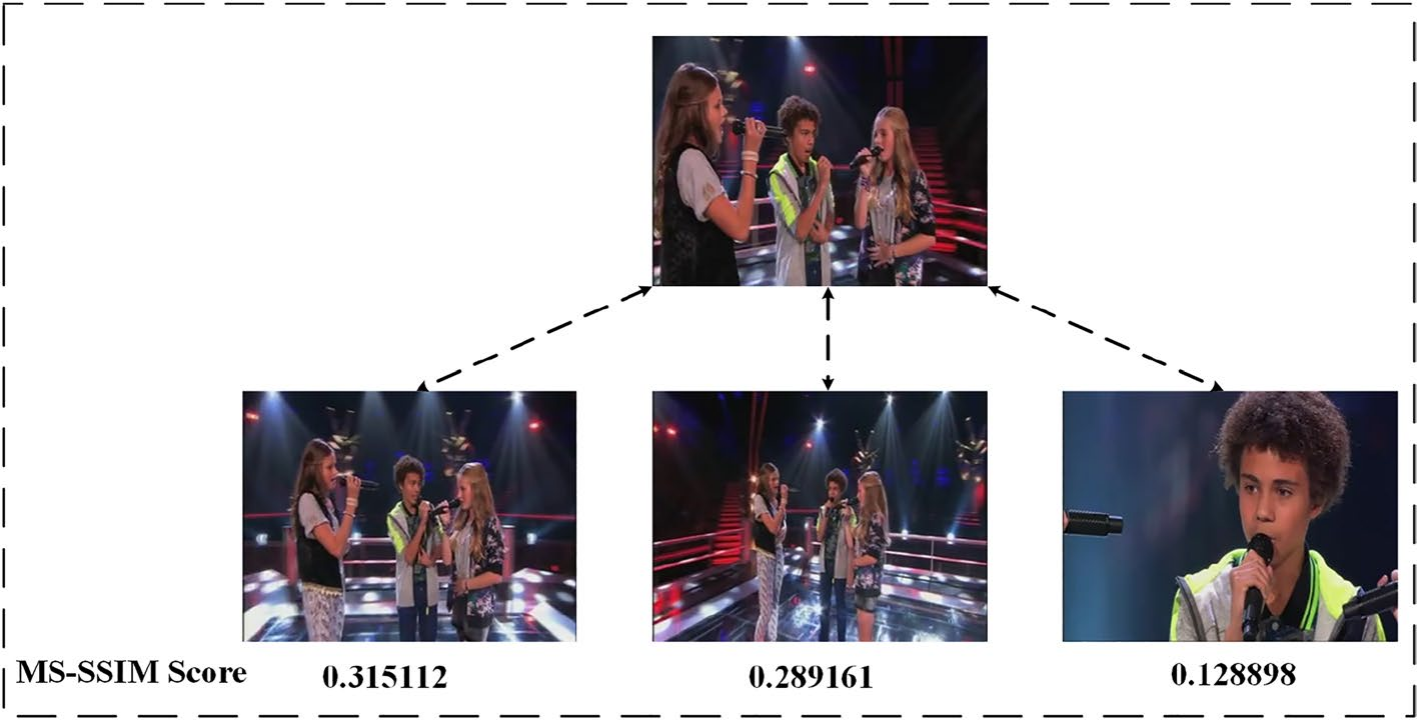
The example of MS-SSIM calculating frame similarity. The plots in the second column are compared with the plots in the first column for similarity to produce the MS-SSIM score. The higher the score the higher the similarity and the lower the score the lower the similarity.

Compared to images, video typically contains more redundant information in consecutive frames, which makes the model more challenging in terms of capacity and computational efficiency. The current mainstream models mainly focus on visual task processing and natural language generation tasks, neglecting the precritical frame selection, which may lead to the degradation of model caption performance. In the field of video keyframe selection, the current mainstream keyframe selection methods^40,41^, involve explicitly choosing keyframes during the video encoding process. A keyframe selection layer is introduced to enhance a regular encoder, predicting the informativeness of each segment based on its contextual representation. While the aforementioned approach has achieved relatively accurate keyframe selection capabilities, the network structure that predicts based on context introduces computational complexity, particularly for longer video sequences, which may lead to higher computational overhead. Therefore, we propose an adaptive scene frame selector in this paper. Firstly, we adopt the MS-SSIM algorithm to compare the multi-scale structural similarities of all frames in the video, which generates similarity scores between two frames as shown in Fig. 2. Secondly, based on the computed scores, representative scene frames are adaptively selected. Then the corresponding keyframes are adaptively selected based on the calculated score as the input frame sequence for the backbone.
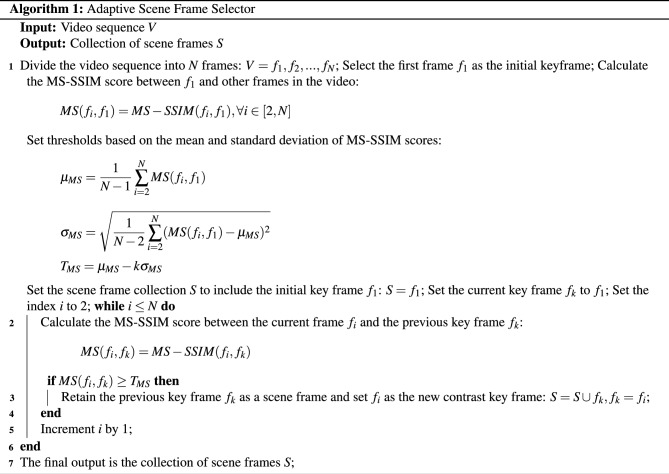


Firstly, the video is segmented into several frames. An initial keyframe is selected and the MS-SSIM score between the initial keyframe and each frame in the video is computed. Then, a threshold is set based on the mean and standard deviation of the MS-SSIM scores. The remaining frames in the video are traversed, and the MS-SSIM score between the current frame and the previous key frame is calculated. If the MS-SSIM score exceeds the set threshold, the current frame is set as a new keyframe. Finally, these steps are repeated until all frames have been processed, and the final output is the key frame sequence. The algorithm is described in Algorithm 1. The original semantic information of the video is preserved in a few frames, and the sampled frames with richer semantic information are obtained to extract visual features.

**Figure 3:**
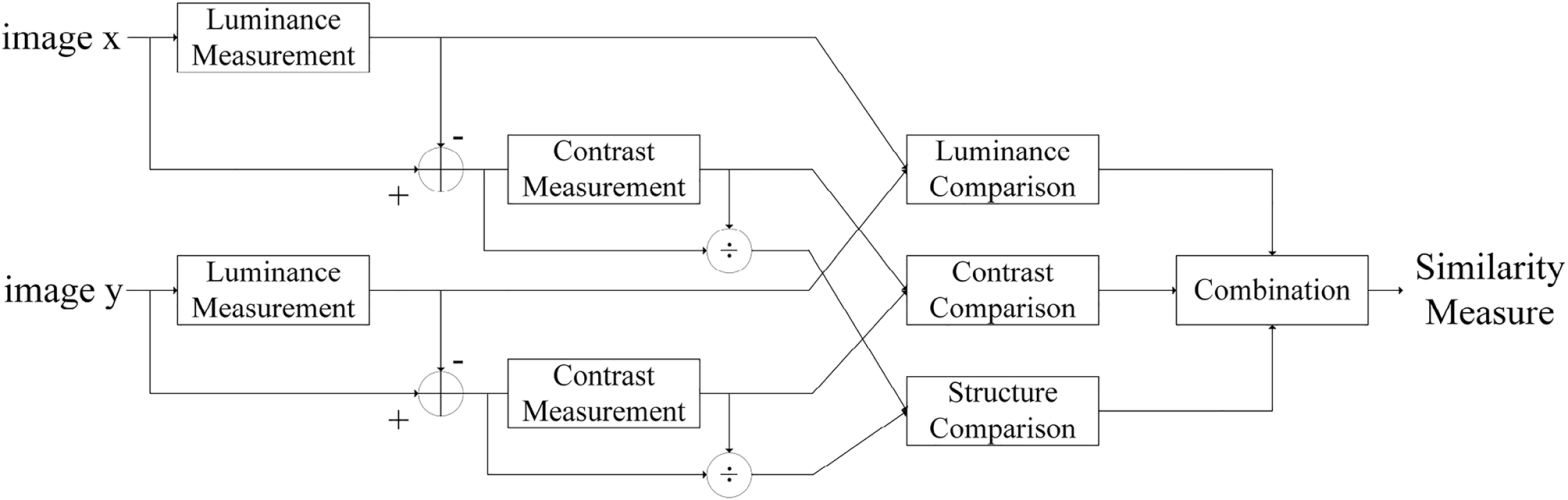
The multiscale structure similarity index measurement system.

The multi-scale structural similarity index metric^42^ (MS-SSIM) is a widely used image quality evaluation metric. The method attempts to quantify the error between distorted images and reference images by using various known characteristics of the human visual system. As shown in Fig. 3, MS-SSIM extracts 3 key features from the image: Brightness, Contrast, and Structure. The two images are compared based on these three features

For images x & y, the SSIM is calculated as follows:

First, calculate the mean of the image:

2 $$\begin{aligned}{} & {} u_{X}=\frac{1}{R \times C} \sum _{i=1}^{R} \sum _{j=1}^{C} X(i, j) \end{aligned}$$

3 $$\begin{aligned}{} & {} u_{Y}=\frac{1}{R \times C} \sum _{i=1}^{R} \sum _{j=1}^{C} Y(i, j) \end{aligned}$$

Calculating the covariance of the image:

4 $$\begin{aligned} {\sigma _{X Y}=\frac{1}{R \times C-1} \sum _{i=1}^{R} \sum _{j=1}^{C}(X(i, j)-u_{X})(Y(i, j)-u_{Y})} \end{aligned}$$

Computing intermediate equations:

5 $$\begin{aligned} \begin{aligned} L(X, Y)&=\frac{2 u_{X} u_{Y}+C_{1}}{u_{X}^{2}+u_{Y}^{2}+C_{1}} \end{aligned} \end{aligned}$$

6 $$\begin{aligned} \begin{aligned} C(X, Y)&=\frac{2 \sigma _{X} \sigma _{Y}+C_{2}}{\sigma _{X}^{2}+\sigma _{Y}^{2}+C_{2}} \end{aligned} \end{aligned}$$

7 $$\begin{aligned} \begin{aligned} S(X, Y)&=\frac{\sigma _{X Y}+C_{3}}{\sigma _{X} \sigma _{Y}+C_{3}} \end{aligned} \end{aligned}$$

where *L(X, Y)* is the luminance contrast factor, *C(X, Y)* is the contrast factor, and *S(X, Y)* is the structure contrast factor.

SSIM calculation:

8 $$\begin{aligned} {\text {SSIM}}(x, y)=[L(X, Y)]^{\alpha } \cdot [C(X, Y)]^{\beta } \cdot [S(X, Y)]^{\gamma } \end{aligned}$$

The formula is generally abbreviated when $\alpha =\beta =\gamma =1$ is taken as:

9 $$\begin{aligned} {\text {SSIM}}(X, Y)=\frac{\left( 2 u_{X} u_{Y}+C_{1}\right) \left( 2 \sigma _{X Y}+C_{2}\right) \left( \sigma _{X Y}+C_{3}\right) }{\left( u_{X}^{2}+u_{Y}^{2}+C_{1}\right) \left( \sigma _{X}^{2}+\sigma _{Y}^{2}+C_{2}\right) \left( \sigma _{X} \sigma _{Y}+C_{3}\right) } \end{aligned}$$

where $u_X$ *is the mean of x*, ${u_y}$ *is the mean of y*, $\sigma _{X}^{2}$ *is the squared difference of x*, $\sigma _{Y}^{2}$ *is the squared difference of y, and* $\sigma _{X Y}$ *is the covariance of x and y*.

SSIM satisfies the following properties.

Symmetry: $${\text {SSIM}}(X, Y)={\text {SSIM}}(Y, X)$$

Boundedness: $${\text {SSIM}}(X, Y) \le 1$$

${\text {SSIM}}(X, Y)=1$ when and only when x and y are the same frame.

It is considered that different receptive fields at different scales can be used to obtain structural similarity, namely MS-SSIM. The input images of our framework as images 1 and 2, and low-pass filter and 1/2 downsampling are used in order. We assume that the original image scale is 1 and the highest scale obtained after *M-1* iterations is *M*. For the *j*-th scale, only the contrast *c(x, y)* and the structural similarity *s(x, y)* are calculated. Luminance similarity *l(x, y)* is calculated only when the scale is *M*. Finally, the results of each scale are concatenated to obtain the MS-SSIM value, as shown in Eq. (10).

10 $$\begin{aligned} {\text {MSSSIM}}(X, Y)=\frac{1}{L} \sum _{i=1}^L w_i \times {\text {SSIM}}\left( x^{(i)}, y^{(i)}\right) ^{g_i} \end{aligned}$$

Where, *x* and *y* represent the input image, *L* represents the scale number of decomposition, $x_i$ and $y_i$ represent the image under the ith scale, $w_i$ represents the weight of the ith scale, and $g_i$ represents the gamma parameter of the ith scale.

### Visual and sentence encoder

In Fig. 1, the model feature extraction stage first takes the keyframes obtained from the adaptive frame sampling module as input to obtain 2 modalities such as images containing static features and video clips with dynamic features. Then, the features are extracted by using two-dimensional convolutional neural networks (2D CNN) and three-dimensional convolutional neural networks (3D CNN) for single-frame modalities and video 3D modalities. Finally, five keyframes were selected and described using the method proposed in reference^43^, and the semantic information represented by each frame was extracted. The word2vec^44^ technique was utilized to embed the semantic information generated from each frame into 300-dimensional vectors. Then, the encoder block of the transformer encoder the acquired semantic and visual information. The output of the encoder block is used as the input to the decoder and finally generates the captions for the video.

*Visual Feature* A large amount of multidimensional information such as objects, scenes, and spatiotemporal relationships exists in the video. Therefore, extracting and fusing this information is crucial for an exact video caption. A migration learning method is used to extract visual features of the target video. The choice of the feature extraction module CNN affects the overall model performance because the encoder receives the CNN features input by the feature extraction module. Therefore, we first analyze the impact of several 2D as well as 3D CNN models most commonly used in the subtitles on the overall model structure. 2D models are selected from ResNet152^45^, VGG^46^, Res-NeXt101^47^, and SE-ResNet152^48^ with the addition of SE module; 3D models are selected from I3D^49^. When comparing different CNN models, the other parts of the model are fixed first, and different CNN models are used for extracting visual features. Then, the features are sent them to the encoder-decoder for video content description generation, and the validation of this part is shown in the ablation experiment section (see subsection “Ablation studies”).

Finally, for the scene information of the corresponding video, the features are extracted and fused using the 2D CNN models SE-ResNet152 and ResNeXt101 networks pre-trained on the dataset ImageNet. The SE-ResNet152 network structure is a combination of the SE Networks in the ResNet152 network structure. In the SE-ResNet152 network architecture, the SE module is applied to the residual branch. The model first reduces the feature dimension to 1/r of the input, uses ReLu for activation, and then boosts the features back to their original dimension through a Fully Connected layer(FC), so that the network has more nonlinearity and better fits complex correlations between channels, while preferably reducing the number of parameters and computational effort. The ResNeXt network is a combination of ResNet and Inception^50^. The network differs from Inception v4 in that ResNeXt does not require the manual design of complex Inception structural details, but instead uses the same topology for each branch. The essence of ResNeXt is Group Convolution, which controls the number of groups by the variable cardinality to achieve a balance between the two strategies. The normalized features extracted from SE-ResNet152 and ResNeXt101 networks are concatenated for feature distance calculation, which is better than using a single model to obtain a better 2D information representation vector.

In processing video temporal motion information, we use the Two-stream Inflated 3D ConvNets (I3D) model pre-trained on the kinetics dataset^51^. First, I3D pre-trains each structure on the Kinetics dataset and then adjusts slightly each structure on the datasets HMDB-51^52^ and UCF-101^53^ to enhance the pretraining performance of the I3D model and enables it to better adapt to downstream tasks.

*Frame Semantics Feature* The video is constructed from multiple static frames in a temporal sequence, so the information generated by describing the content of each scene keyframe selected through an adaptive frame selector in the video contributes to the overall video content description. The sampled frames are captioned using an image caption generation system^43^ pre-trained on the authoritative Microsoft Common Objects in Context (COCO) dataset, and then the corresponding subtitle text is converted to a 300-d vector representation using the word2vec model. The literature^54^ also states that Word2vec trains word vectors according to context information, so the word vectors trained by the word2vec model contain context semantic information.

*Vision Transformer Encoder* Conventional encoder and decoder models are usually RNN structures, but there are two problems in the sequential calculation of RNN series models. a) Each time state is dependent on the previous time step state, which results in the model not being able to be accelerated by parallel computing. b) A series of models in the RNN series, such as GRU and LSTM, have introduced a gate mechanism. But they have the disadvantage of a limited ability to handle long-term dependency problems. Inspired by the success of Transformer^55^ on a wide range of tasks in NLP, Vision Transformer (ViT)^56^ is the first to adopt a pure transformer architecture for image classification, which shows the good performance of transformer architecture on vision tasks. Therefore, we also use the transformer encoder as our model encoder when designing the model structure, which can effectively use the intermediate state of the transformer encoder to achieve global encoding of video features.

**Figure 4:**
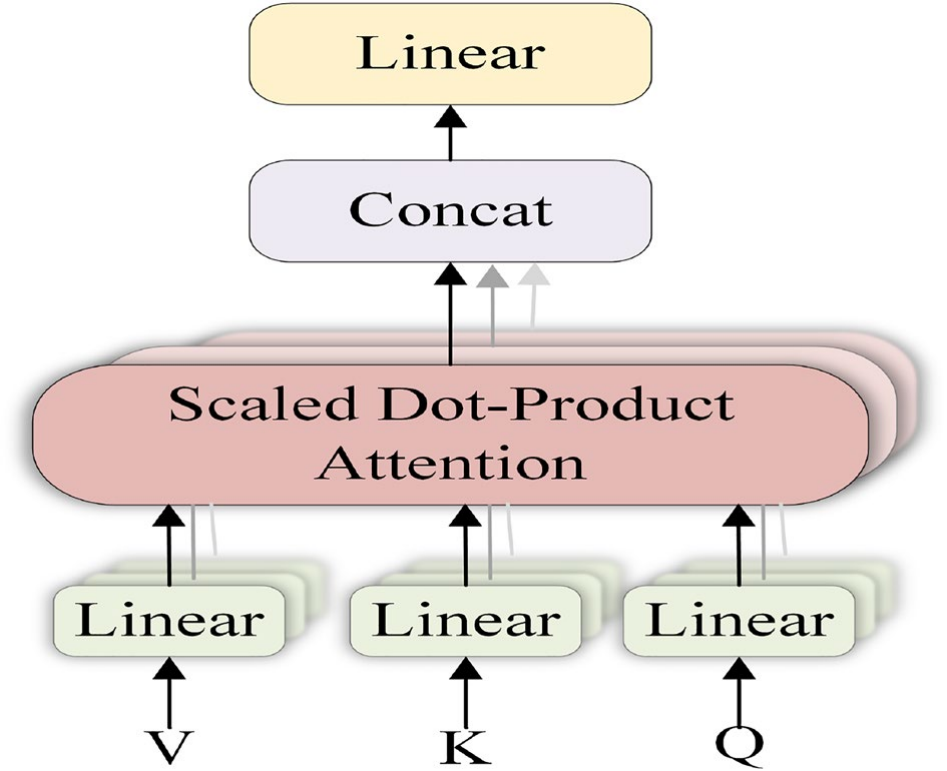
Multi-head attention mechanism. Q, K, and V represent three matrices. Three Linear layers and Scaled Dot-Product Attention layers represent three heads. Then the output of the three heads of attention is concatenated. Finally, the output of the concat operation is transformed into the same output value as that of a single head by the linear layer.

The model encoder sets up a stack of 12 encoder blocks, each of which contains two sub-layers: Interactive Multi-Head Attention (MSA) and Multilayer Perceptron (MLP). The multi-head attention structure is shown in Fig. 4. In this structure, the original vector is insidiously projected into multiple low-dimensional spaces through the linear layer, and then the Scaled Dot-product Attention (SDP Attention) operation is performed in parallel, and then spliced, which can achieve better results. As shown in Eqs. (11–12). Map an input vector into multiple lower-dimensional vectors first, which is equivalent to dividing it into multiple perspectives. Then attention is performed at each perspective to improve the learning ability of the model. This structure parameterizes the dimensionality reduction process so that the model can efficiently learn the most useful perspective from the data.

11 $$\begin{aligned} {\text {head}}_{\textrm{i}}={\text {Attention}}\left( Q W_{i}^{Q}, K W_{i}^{K}, V W_{i}^{V}\right) \end{aligned}$$

12 $$\begin{aligned} \text{ MultiHead }(Q, K, V)=\text {Concat}\left( \text {head}_{1}, \ldots , \text{ head}_{\textrm{h}}\right) W^{O} \end{aligned}$$

At the *l*-th time step shown by the encoder module structure in Fig. 1 (model structure), the video feature extracted by the convolutional network is $x_v$. First, the MSA is used to calculate the normalized previous time step feature as $x_{l-1}$. Then, the output of the coded block is calculated using the MLP function. Finally, its *N* features are normalized as the final feature representation of the encoder. The output size of this encoder is 1024-d. The calculation process is shown in Eqs. (13–15).

13 $$\begin{aligned} z_{l}^{\prime }= & {} M {\text {SA}}\left( L N\left( z_{l-1}\right) \right) +z_{l-1} \quad l=1 \ldots N \end{aligned}$$

14 $$\begin{aligned} z_{l}= & {} M {\text {LP}}\left( {\text {LN}}\left( z_{l}^{\prime }\right) \right) +z_{l}^{\prime } \quad l=1 \ldots N \end{aligned}$$

15 $$\begin{aligned} x_{l}= & {} L N\left( z_{l}\right) \quad l=1 \ldots N \end{aligned}$$

where $z^{'}_l$ represents the output of the multi-head attention mechanism; $z_l$ is the output of the multi-layer perceptron; $x_l$ is the output of the encoder at time *l*, which can also be expressed as the result of global feature encoding; *N* is the total time step length.

### Captioning generator

**Figure 5:**
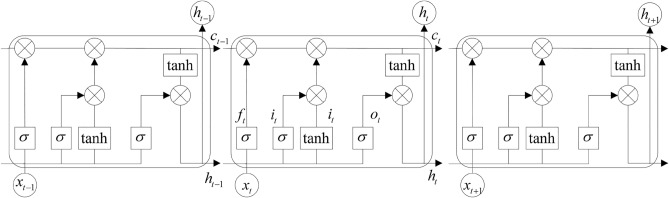
The LSTM network structure.

LSTM, as a sequential model, progressively generates output sequences. In the context of video captioning, the generation of descriptive sentences typically involves a temporal sequentiality, where frames are generated in accordance with chronological order. LSTM demonstrates prowess in handling such sequential generation tasks. In contrast, Transformer’s self-attention mechanism may lead to heightened computational complexity during the decoding phase, particularly when generating longer sequences. In comparison, LSTM presents relatively diminished computational overhead, thereby rendering it more efficient in the decoding process. This efficiency assumes an advantageous role when grappling with extensive video datasets and lengthy sequences. Given the pronounced temporal nature of videos and the underlying sequential dependencies among corresponding natural language descriptors and words, we observe that our generated captions remain relatively succinct, obviating the necessity for handling extensive dependencies. Consequently, the decoder module of our model adopts a multi-layered architecture of Long Short-Term Memory (LSTM) networks, which serve as the foundational structures for the decoding process. The LSTM unit network structure is shown in Fig. 5, which consists of components such as an input gate, forget gate, output gate, and memory unit.

At time *t*, it is assumed that the model encoding output is a 1024-d vector $x_t$, the output hidden layer result of the LSTM gating unit in the previous step is $h_{t-1}$, and the memory unit in the current step is $c_t$. We use the activation function $\sigma$ to weight the feature vector to obtain the output of the input gate as the input feature vector $i_t$ of the LSTM unit. Similarly, the forget gate feature ft and the output feature $o_t$ can be obtained. The calculation is shown in Eqs. (16–21).

16 $$\begin{aligned} i_{t}= & {} \sigma \left( W_{x i} x_{t}+W_{\textrm{hi}} h_{t-1}+b_{l}\right) \end{aligned}$$

17 $$\begin{aligned} f_{t}= & {} \sigma \left( W_{x f} x_{t}+W_{\textrm{hf}} h_{t-1}+b_{f}\right) \end{aligned}$$

18 $$\begin{aligned} o_{t}= & {} \sigma \left( W_{x o} x_{t}+W_{\textrm{ho}} h_{t-1}+b_{o}\right) \end{aligned}$$

19 $$\begin{aligned} g_{t}= & {} \phi \left( W_{x g} x_{t}+W_{\textrm{lg}} h_{t-1}+b_{g}\right) \end{aligned}$$

20 $$\begin{aligned} c_{t}= & {} f_{t} \odot c_{t-1}+i_{t} \odot g_{t} \end{aligned}$$

21 $$\begin{aligned} \mathrm {~h}_{t}= & {} o_{t} \odot \phi \left( c_{t}\right) \end{aligned}$$

where $\sigma$ is the sigmoid activation function and $\phi$ is the tanh activation function. $\odot$ is the Hadamard product operation.

We obtain the corresponding hidden layer output $H=(h_1$,h$_2$, h$_3$,..., h$_n$,) by taking the encoded feature sequence as the input of the decoder LSTM network in the video content captioning decoding process. After the model decoder inputs the last feature vector, the output result of the hidden layer of the LSTM network is the decoding result of the current video frame sequence. The LSTM decoder is shown in Algorithm 2.
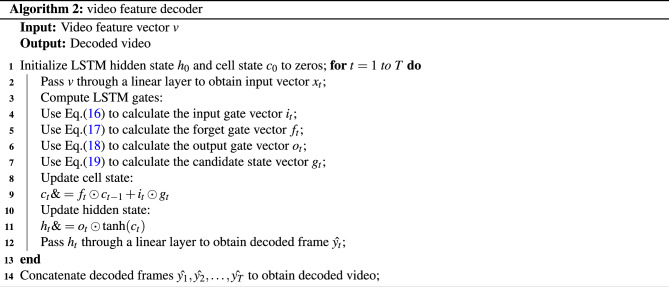


### Nonparametric metric learning module

In this section, nonparametric metric learning is used to reduce the discrepancy between model-generated and ground-truth captions. By comparing the captions generated by the model with the ground truth, the vector representations of the generated captions are semantically similar to the ground truth.

There are two sequences in the metric learning module: the video captioning sequence generated by the model and the real data video captioning sequence. We employ an LSTM network structure to embed sentence sequences into a sentence-vector model. Each of these LSTMs has 1024d hidden and output layers. Furthermore, a cosine similarity loss^57^ is utilized to compare two 1024d vectors $S_1$ and $S_2$ corresponding to the generated and real captions. When using the cosine angle between vectors to measure similarity, the smaller the angle between vectors, the higher the similarity between texts. An angle with a cosine angle greater than 90 degrees means that the vectors are orthogonal to each other, which indicates that the sentences are not related to each other. The cosine similarity between two sentences can be expressed as Eq. (22).

22 $$\begin{aligned} L_{s i m}=\frac{{\textbf{S}}_{1} \cdot {\textbf{S}}_{2}}{\left\| {\textbf{S}}_{1}\right\| \left\| {\textbf{S}}_{2}\right\| }=\frac{\sum _{i=1}^{n} {\textbf{S}}_{1 i} {\textbf{S}}_{2 i}}{\sqrt{\sum _{i=1}^{n}\left( {\textbf{S}}_{1 i}\right) ^{2}} \sqrt{\sum _{i=1}^{n}\left( {\textbf{S}}_{2 i}\right) ^{2}}} \end{aligned}$$

where $S_1$, $S_2$ is the dot product of two sentences, divided by the product magnitude of two sentences $\Vert S_1 \Vert$, $\Vert S_2 \Vert$.

It is found that if only a real description is randomly selected as the calculation vector, the model will give a generation description with a great difference at the beginning of training, and the network will learn a mapping very similar to the extracted ground truth. For $S_1$ and $S_2$, the $L_{sim}$ loss quickly becomes small. To prevent this, we adopt more different caption pairs in the measurement process. Because a video in the dataset contains many real descriptions and different real captions can be regarded as different sentences. We hence can sample different caption pairs in $L_{sim}$ batches to compute an average similarity loss *L* as shown in Eq. (23), which can improve the robustness of the model generation description.

23 $$\begin{aligned} L=\root n \of {L_{sim1} \cdot L_{sim2} \cdots L_{simn}}=\root n \of {\prod _{\textrm{i}=1}^{n} L_{sim_{i}}} \end{aligned}$$

## Experiments

### Datasets

Here, three commonly used benchmark datasets: MSVD^58^, MSR-VTT^39^, and LSMDC^59^ are utilized to assess the final effectiveness of our method. To standardize the datasets, the length of the sentence is set to 20 words, add the tags <BOS> and <EOS> to the beginning and end of the sentence, and the sentences with a length of fewer than 20 words is set to zero in the end.

*MSVD* The YouTube2Text dataset, also known as the MSVD dataset, contains 1970 videos collected, covering different events. Each video contains about 40 sentence tags, which is suitable for training video subtitle methods. The number of training sets, verification sets, and test sets is 1200, 100, and 670 respectively.

*MSR-VTT* MSR-VTT is a widely used public dataset published by Microsoft for use in video-generated text research in 2016. We use the updated version of MSR-VTT released in 2017. The dataset contains 10000 training video clips and 3000 test video clips, with a total time of 41.2 hours. On average, each segment contains 20 natural language annotation statements, with a total of 200000 statements. The dataset consists of 257 popular categories from 20 representative classes of real video search engines, which is conducive to enhancing and verifying the generalization ability of the video semantic description algorithm.

*LSMDC* Large Scale Movie Description Challenge LSMDC(https://sites.google.com/site/describingmovies/download) is presented based on the joint presentation of the MPII Movie Description Dataset (MPII-MD) and the Montreal Video Annotation Dataset (MVAD). The dataset consists of 128k sentence fragment pairs and 158h of the video. The training, validation, public, and blind test sets contain 100000, 1000, 1000, and 1000 video clips, respectively. Among them, the blind test set does not give the standard description sentence in advance, so the results need to be submitted to the CodaLab website, and the website will give the evaluation score.

### Evaluation metric

We adopt four algorithms widely used in the video captioning field, CIDEr(Consensus based Image Description Evaluation)^60^, METEOR(Metric for Evaluation of Translation with Explicit Ordering)^61^ ROUGE-L(Recall Oriented Understudy for Gisting Evaluation Longest Common Subsequence)^62^ and BLEU (Bilingual Evaluation Understudy)^63^ are used as evaluation metric to calculate evaluation scores for our model and comparison model, to objectively evaluate the statement description generation effect of the final model.

### Implementation details

*Vocabulary Preprocessing* For each sentence, we first segment the sentence using the Stanford NLP Toolkit(https://stanfordnlp.github.io/CoreNLP/) and truncate it into 20 words, and then remove punctuation and convert each word to lowercase. In order to form vocabularies, the minimum occurrence frequency of each word is set as 1, which ultimately resulted in a vocabulary of 7,184 words for the MSVD and 16,860 words for the MSRVTT. To specific, random initialization is used to embed words.

*Feature Extraction* When extracting features, each frame is first randomly rotated by $15^{\circ }$ and then randomly cropped to obtain an image of the $224\times 224$ pixel size. The 2048d feature vectors extracted by 2D CNN network SE_ResNet152 and ResNeXt101 are fused into 4096d image features. Secondly, the sampled frame is used to the input of 3D CNN network model I3D according to temporal orders, and features are extracted on the RGB and optical flow I3D inputs respectively. The output of the MIXED_5C layer of the I3D network structure is used as the spatiotemporal features, and the 2048-d image 3D features are finally fused. Then, five frames are selected as the input of the static image caption model on the COCO dataset at medium intervals in the adaptive sampling frameset, and five corresponding text descriptions are output, and the corresponding descriptions are embedded into a 300-d vector using the word2vec model.

*Encoder-decoder* Before inputting the video features into the model, the visual features and semantic features are mapped into 1024-d vectors respectively, and the words are embedded into 300-d vectors.

In the feature extractor, firstly, we represent the video as a sequence of images ${I_1, I_2, \ldots , I_T}$, where *T* denotes the total number of frames in the video. For each image $I_t$, we extract its semantic features using 2D CNN, 3D CNN, and word2vec. Specifically, the features extracted using 2D CNN, 3D CNN, and word2vec are denoted as $f_t^{2D}$, $f_t^{3D}$, and $f_t^{W2V}$, respectively. We then fuse these features into a single feature vector $f_t$ for each frame *t* by concatenating them along the channel dimension, $f_t$ as shown in Eq. (24). We stack these frame feature vectors to form a video feature representation ${\textbf{v}} \in {\mathbb {R}}^{T \times C}$, as shown in Eq. (25). Finally, we use a Vision Transformer as the encoder to map the input sequence ${\textbf{v}}$ to a set of high-dimensional feature vectors ${\textbf{z}} = {{\textbf{z}}_1, {\textbf{z}}_2, \ldots , {\textbf{z}}_T}$, where each ${\textbf{z}}_t$ is computed as follows Eq. (26).

24 $$\begin{aligned} {{\textbf{f}}_t = [f_t^{2D}, f_t^{3D}, f_t^{W2V}] \in {\mathbb {R}}^{C}} \end{aligned}$$

where *C* is the total number of channels in all the features.

25 $$\begin{aligned} {\textbf{v}}= & {} [f_1; f_2; \ldots ; f_T] \in {\mathbb {R}}^{T \times C} \end{aligned}$$

26 $$\begin{aligned} {\textbf{z}}_t= & {} \text {ViT}({\textbf{v}})_t \end{aligned}$$

Here, $\text {ViT}(\cdot )$ represents the encoding process of the Vision Transformer, and $_t$ denotes the encoding at the *t*-th position in the sequence. The Vision Transformer consists of multiple Transformer encoding blocks and uses the default implementation.

In the model training phase, the Adam^64^ is experimentally applied to optimize the proposed model, and the initial value of this optimizer parameter is $\alpha =0.9$, $\beta =0.999$, $\varepsilon =10^{-8}$. The initial learning rate is set to 0.0001, which is continuously reduced with the training iterations. In order to prevent the model from overfitting, the auto dropout strategy proposed by Pham et al.^65^ is used in this paper to achieve regularization. The initial values of all the weights to be gradient updated are set to be uniformly distributed on the interval [-0.08, 0.08]. The set search size for the model testing phase is set as 5.

*Cross-entropy loss* In this paper, the cross-entropy loss function is applied to calculate the deviation of the target value from the actual output value. The optimal value intervals used for positive and negative samples are obtained through extensive experiments. This function turns the output of the neural network into a probability map. From this, the cross-entropy can be used to compute the distance between the predicted probability map and the actual output value. Meanwhile, the inclusion of the equilibrium parameter $\theta = 1.1$ can improve the prediction accuracy. The calculation is shown in Eqs. (27–28).

27 $$\begin{aligned} X_{t}=w h_{t}+b \end{aligned}$$

where $h_t$ is the decoder output hidden vector. $x_t$ is the fully connected result.

28 $$\begin{aligned} P(y \mid x)=\frac{e^{h\left( x, y_{i}\right) }}{\sum _{j=1}^{n} e^{h\left( x, y_{i}\right) }} \end{aligned}$$

where *x* presents the fully connected result, *y* indicates the ground truth, and *P* is the softmax.

When incorporating the balance parameter into the cross-entropy loss function, the impact of different types of samples can be adjusted by assigning different weights to positive and negative samples. Suppose there are n samples, where the true label and predicted probability of the i-th sample are denoted as $y_i$ and $p_i$, respectively. Let alpha be the balance parameter, w be the weight of positive samples, and 1-w be the weight of negative samples. The cross-entropy loss function with balance parameter can be defined as Eq. (29).

29 $$\begin{aligned} {L(\theta ) = -\frac{1}{n} \sum _{i=1}^{n} \left[ \alpha y_i w \log p_i + (1-\alpha ) (1-y_i) (1-w) \log (1-p_i) \right] } \end{aligned}$$

where $\theta$ represents the model parameters.

### Experimental results and comparison

**Figure 6:**
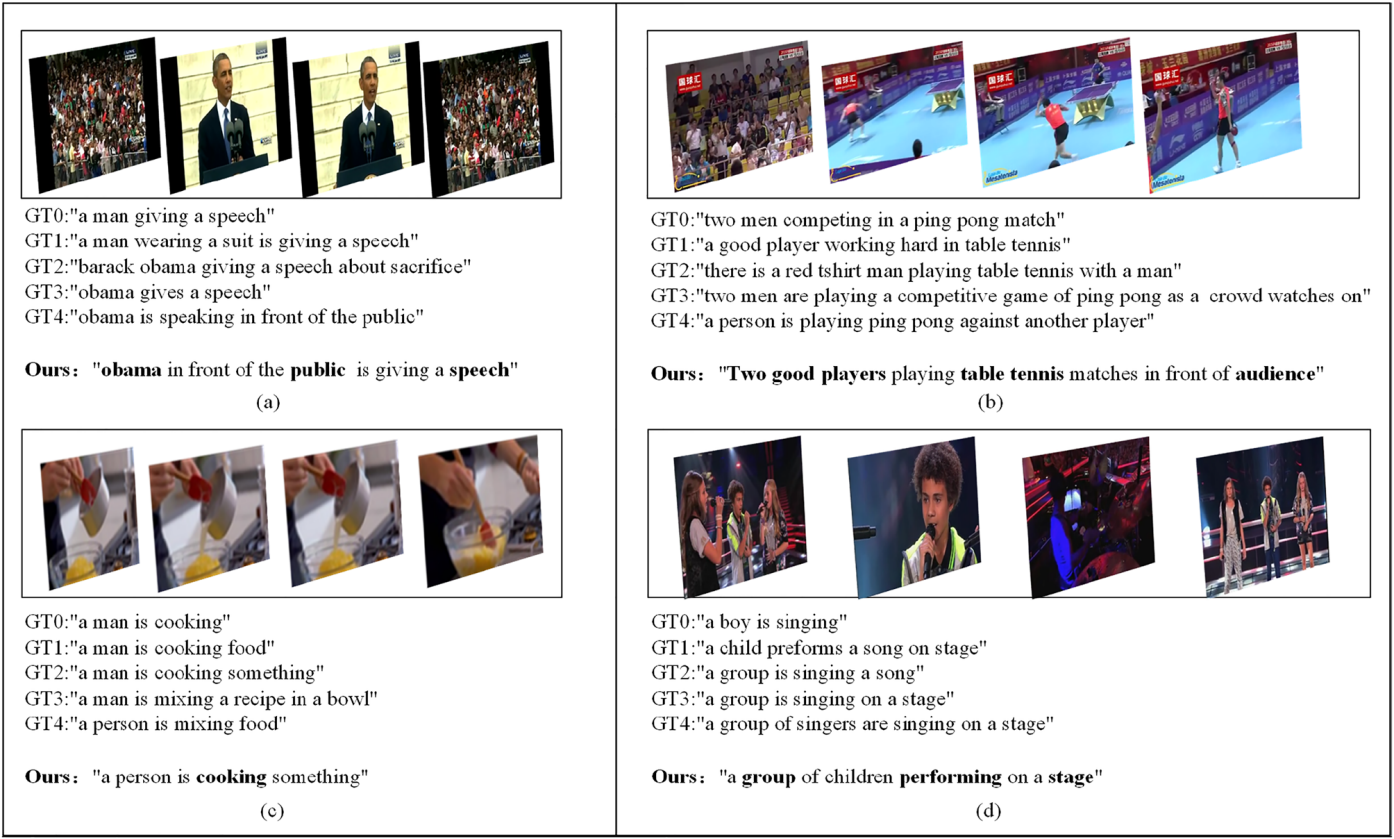
Comparison of visualization of generated description examples. where GT stands for ground truth and ours stands for our optimal model test results.

To further verify the performance of the proposed method, it is evaluated on the MSVD, MSR-VTT, and LSMDC datasets, respectively. VC-HRNAT^66^, SGN^38^, SCST^67^, STG^68^, SAAT^33^, E2E^69^, and POS-CG^70^ are used as competitor models. The qualitative results can be found in Tables 1,  2, and 3, and the quantitative results can be seen in Fig. 6. B4, M, R, and C are used to denote the evaluation indicators BLUE-4, METEOR, ROUGE-L, and CIDEr-D, respectively.

It can be seen from Table 1, the proposed method can obtain 42.20 BLEU4, 28.90 METEOR, 62.16 ROUGE-L, and 54.30 CIDEr-D under the MSR-VTT dataset. In particular, in the METEOR metric, compared to the two best-performing models among all compared models, SCST and SGN, the model in this paper can improve 0.34% (28.80 vs 28.90) and 2.12% (28.30 vs 28.90), respectively; in the ROUGE-L metric, for the two best-performing models in this metric, VC-HRNAT (IR+C) and POS-CG (IR+M), the model in this paper can improve 0.74% ( 61.70 vs 62.16) and 0.91% (61.60 vs 62.16), respectively; in the CIDEr-D indicator, the model in this paper can improve 0.36% (54.10 vs 54.30) and 6.47% (51.00 vs 54.30) respectively compared with the SCST and SAAT models, and the increase of the CIDEr-D indicator is more significant, which indicates that semantic-guided learning has a positive effect on generating the final description as a whole.

Furthermore, we further conduct and analyze comparative experiments on the MSVD benchmark dataset to validate the performance of our method. The detailed results can be found in Table 2, and it is observed that the proposed method under the benchmark dataset MSVD can obtain 55.3 BLEU4, 36.1 METEOR, 74.2 ROUGE-L, and 98.4 CIDEr-D. Specifically, in the CIDEr-D metric, our model compared to the best performing VC-HRNAT(IR+I) and STG models among all comparison models can improve by 0.30% (98.1 vs 98.4) and 5.81% (93.0 vs 98.4), respectively.

**Table 1:** Different model results on a test set of the MSR-VTT dataset.

Years	Method	B4	M	R	C
2022	vc-HRNAT(IR+C)^66^	**43.00**	28.20	61.70	49.60
2022	vc-HRNAT(IR+I)^66^	42.10	28.00	61.60	48.20
2021	SGN^38^	40.80	28.30	60.80	49.50
2021	SCST^67^	40.30	28.80	61.20	54.10
2020	STG^68^	40.50	28.30	60.90	47.10
2020	SAAT^33^	39.90	27.70	61.20	51.00
2019	E2E^69^	40.40	27.00	61.00	48.30
2019	POS-CG(I3D+M)^70^	41.70	27.80	61.20	48.50
2019	POS-CG(IR+M)^70^	42.00	28.20	61.60	48.70
	**Ours**	42.20	**28.90**	**62.16**	**54.30**

The best experimental results are presented in bold.

**Table 2:** Different model results on the testset of the MSVD dataset.

Years	Method	B4	M	R	C
2022	vc-HRNAT(IR+C)^66^	**57.7**	36.3	74.0	96.3
2022	vc-HRNAT(IR+I)^66^	55.7	**36.8**	74.1	98.1
2021	SGN^38^	52.8	35.5	72.9	94.3
2021	SCST^67^	50.9	35.1	72.4	94.5
2020	STG^68^	52.2	36.9	73.9	93.0
2020	SAAT^33^	46.5	33.5	69.4	81.0
2019	E2E^69^	50.3	34.0	70.8	87.5
2019	POS-CG(I3D+M)^70^	53.5	34.9	72.1	91.0
2019	POS-CG(IR+M)^70^	52.5	34.1	71.3	88.7
	**Ours**	55.3	36.1	**74.2**	**98.4**

The best experimental results are presented in bold.

Table 3 shows some comparisons between the results of the proposed method and the competitors in this contest on the LSMDC dataset. We observe that although there is a gap between the proposed model and the most advanced model on the METEOR index, the model obtains the best results on the index BLEU4, ROUGE-L, and CIDEr-D. The score of 0.018, 0.170, and 0.166 is obtained under the movie description contest dataset LSMDC. Compared with the results of the first player, on the metric BLEU4, ROUGE-L, and CIDEr-D. ROUGE-L and CIDEr-D increased by 63.63%, 5.59%, and 50.91%, respectively.

From Tables 2 and  3, it can be seen that our proposed model performs well in multiple metrics. However, it falls slightly behind the best-performing baseline model in the METEOR metric. Upon analysis, it is known that the METEOR metric is based on the measurement method of weighted harmonic mean and unigram recall rate. METEOR mainly focuses on whether the translated results are semantically similar, rather than considering the consistency of syntax and structure. In order to improve the grammaticality of the generated descriptions, our proposed model adopts metric learning to optimize the generated sentences. Consequently, this results in METEOR being less accurate in evaluating syntax and structure, potentially leading to inaccurate scores.

The above results suggest that our model not only performs well on the mainstream MSR-VTT and MSVD datasets but also achieves good performance on the practical application dataset LSMDC, which further verifies the generalization performance of the model in this paper. In addition, it can be seen from Fig. 6 that the descriptions generated by the proposed model can accurately express the real content of the video and fit the natural language expression.

**Table 3:** Quantitative comparison with participant results on the LSMDC dataset.

Users	B4	M	R	C
ElTanque	0.007	0.055	0.139	0.110
Danieljf24	0.011	**0.070**	0.160	0.107
Arohrbach	0.009	**0.070**	0.161	0.106
YueHu	0.003	0.061	0.159	0.077
Phyllis	0.004	0.065	0.156	0.076
**Ours**	**0.018**	0.067	**0.170**	**0.166**

The best experimental results are presented in bold.

### Ablation studies

The performance of the video content understanding model relies on the backbone of CNN. In order to verify the effectiveness of different parts in our model, the performance of the model is compared in different scenarios by modifying or removing a part of the model, or changing the training strategy. In the two different cases where the encoder uses the conventional LSTM and the Encoder Block of the Vision Transformer architecture, six different backbone combination architectures are performed separately for model performance testing, namely VGG, ResNet152, SE_ResNet152, VGG combines with ResNeXt101, ResNet152 combines with ResNeXt101, SE_ResNet152 combines with ResNeXt101, and the 3D, as well as optical flow features, are extracted by choosing the I3D structure fixed.

The difference between using and not using metric learning in model training lies in the optimization objective of the model during training. In traditional model training without metric learning, the objective is typically to minimize a loss function, such as cross-entropy loss. In this case, the model is trained to directly predict the class or probability distribution of the samples. In contrast, in metric learning-based model training, the objective is to learn a distance or similarity function that can map similar samples to nearby feature space. In this case, the model is trained to predict the similarity between samples, rather than directly predicting their class or probability distribution. This approach is typically used for learning low-dimensional embeddings for easier data visualization and analysis.

Analyzing the results of ablation experiments, it can be observed that models trained using metric learning can have better generalization performance, as they consider the similarity between samples rather than just their class labels. Additionally, metric learning can handle data in non-Euclidean spaces, such as text and images.

### Comparison of the different video feature extraction architectures

To better quantify the impact of different backbones on our model performance, several ablation studies are conducted to investigate the performance variation due to different backbone structures. It is noticed that each backbone ablation model refers to fixing other components and changing only the feature extraction module, and all ablation experiments are performed on the same dataset segmentation and training settings, and the results of the MSR-VTT benchmark dataset experiments are shown in Tables 4 and 5. The results of the ablation experiments on the MSVD benchmark dataset are shown in Tables 6 and 7. Experiments on MSVD and MSR-VTT datasets show that when other components are fixed, such as fixed encoder, the quantitative results of the LSTM network and ViT Encoder Block are shown in Tables 4, 5, 6, and 7. Comparing the experimental results in the above Tables, it can be found that for the selection of CNN structure, when the features extracted from two 2D CNN network structures, SE-ResNet152 and ResNeXt101, are combined as 2D features, and the fused features of 3D and optical flow features extracted from I3D structure as video vision feature extractor can make the model experimental results further improved.

The SE-ResNet152 introduces a channel attention mechanism called Squeeze-and-Excitation, which improves the model’s focus on important features by learning the importance weights of each channel. On the other hand, ResNeXt101 uses group convolution to increase the model’s width without increasing its computational complexity, which enhances the network’s expressive power. In this work, we propose a model that combines the features extracted from both SE-ResNet152 and ResNeXt101 as 2D feature representations. Our experiments show that compared to using a single 2D CNN, the training time of the proposed model increases by approximately 10% to 12% due to the use of fused features.

**Table 4:** Quantitative results of different backbone combinations without metric learning and using LSTM or ViT Encoder Block as encoder architecture under the MSR-VTT benchmark dataset.

Backbones					Score							
VGG	ResNet152	SE_ResNet152	ResNeXt-101	I3D	B4		M		R		C	
					LSTM	ViT	LSTM	ViT	LSTM	ViT	LSTM	ViT
$\checkmark$				$\checkmark$	37.50	36.92	27.90	26.92	58.61	58.30	41.12	42.28
$\checkmark$			$\checkmark$	$\checkmark$	37.90	41.30	26.93	27.15	56.92	58.89	42.09	42.74
	$\checkmark$			$\checkmark$	38.40	34.89	27.30	27.80	58.46	59.82	44.73	50.49
	$\checkmark$		$\checkmark$	$\checkmark$	39.90	42.00	28.61	26.60	59.70	61.49	50.22	50.20
		$\checkmark$		$\checkmark$	39.50	38.65	27.33	27.10	61.20	61.00	50.20	51.00
		$\checkmark$	$\checkmark$	$\checkmark$	41.20	41.70	27.40	27.31	61.20	61.30	51.08	52.20

**Table 5:** Quantitative results of different backbone combinations using metric learning and LSTM or ViT Encoder Block as encoder architecture under MSR-VTT benchmark dataset.

Backbones					Score							
VGG	ResNet152	SE_ResNet152	ResNeXt-101	I3D	B4		M		R		C	
					LSTM	ViT	LSTM	ViT	LSTM	ViT	LSTM	ViT
$\checkmark$				$\checkmark$	38.20	38.60	27.70	28.00	59.10	59.50	43.80	44.80
$\checkmark$			$\checkmark$	$\checkmark$	39.00	40.80	27.82	28.25	59.10	60.20	44.20	45.70
	$\checkmark$			$\checkmark$	39.50	39.20	28.10	27.90	60.30	60.90	46.80	52.50
	$\checkmark$		$\checkmark$	$\checkmark$	40.80	41.50	28.40	28.60	61.20	62.00	52.42	51.80
		$\checkmark$		$\checkmark$	39.10	40.50	27.90	28.20	60.90	61.50	52.20	52.00
		$\checkmark$	$\checkmark$	$\checkmark$	41.50	42.20	28.40	28.90	61.80	62.16	52.80	54.30

**Table 6:** Quantitative results of different Backbone combinations without metric learning and using LSTM or ViT Encoder Block as encoder architecture under MSVD benchmark dataset.

Backbones					Score							
VGG	ResNet152	SE_ResNet152	ResNeXt-101	I3D	B4		M		R		C	
					LSTM	ViT	LSTM	ViT	LSTM	ViT	LSTM	ViT
$\checkmark$				$\checkmark$	52.9	53.5	35.0	35.0	71.9	70.8	92.3	93.9
$\checkmark$			$\checkmark$	$\checkmark$	52.6	54.0	34.3	34.9	72.6	73.6	94.8	95.0
	$\checkmark$			$\checkmark$	54.7	53.5	34.8	35.0	72.9	72.3	93.2	94.2
	$\checkmark$		$\checkmark$	$\checkmark$	54.0	55.1	35.1	34.2	72.0	72.0	94.5	95.9
		$\checkmark$		$\checkmark$	52.1	53.2	35.0	33.6	70.9	73.5	96.0	96.6
		$\checkmark$	$\checkmark$	$\checkmark$	54.8	54.6	33.9	35.8	73.0	72.9	96.3	97.0

**Table 7:** Quantitative results of different backbone combinations using metric learning and LSTM or ViT Encoder Block as encoder architecture under MSVD benchmark dataset.

Backbones					Score							
VGG	ResNet152	SE_ResNet152	ResNeXt-101	I3D	B4		M		R		C	
					LSTM	ViT	LSTM	ViT	LSTM	ViT	LSTM	ViT
$\checkmark$				$\checkmark$	53.3	53.9	35.2	35.0	71.8	72.4	93.7	94.2
$\checkmark$			$\checkmark$	$\checkmark$	54.6	55.1	35.0	35.3	73.2	74.0	94.8	95.8
	$\checkmark$			$\checkmark$	55.0	54.3	34.1	34.9	72.6	73.1	94.8	95.5
	$\checkmark$		$\checkmark$	$\checkmark$	54.3	55.0	35.2	35.6	72.0	74.0	95.0	96.1
		$\checkmark$		$\checkmark$	54.0	54.9	34.0	34.4	71.6	73.8	95.9	95.9
		$\checkmark$	$\checkmark$	$\checkmark$	54.8	55.3	35.8	36.1	73.3	74.2	97.5	98.4

*Effect of encoder on model performance* Current model frameworks for deep learning video caption generation are typically designed as encoder-decoder architectures, in which the encoder learns a compressed representation of the video from multimodal features, and the decoder uses the video representation output from the encoder to generate a sentence word-by-word. The learning of video encoder representations is the basis of the video understanding, so the quantitative results of the ablation experiments with different encoder architectures while fixing other model structures are shown in Tables 8 and 9. In this paper, the encoder part of the model structure is consisted of transformer encoder blocks by using the global view to learn the features hard-coded into CNN, reduce the loss of information in the hidden layer in the middle of the encoder, and freely learn the mixture of local and global features in the lower layer, which is helpful to enhance the generalization ability of the proposed model.

*Impact of metric learning modules on the model structure* In order to enhance the accuracy of the proposed model, a metric learning method based on cosine similarity is introduced. The quantitative results of its metric validity test are shown in Table 10. These results illustrate that two benchmark datasets MSR-VTT and MSVD are improved under evaluation metrics BLEU-4, METEOR, ROUGE-L, and CIDEr-D in the case of using the metric learning module, and the CIDEr-D metric rise more significantly.

**Table 8:** Quantitative results of different encoders without metric learning in fixed Backbone network under MSR-VTT and MSVD benchmark dataset.

Backbones	Dataset	Encoder	Score			
			B4	M	R	C
SE_ResNet152+ResNeXt-101+I3D	MSR-VTT	LSTM	41.20	27.40	61.20	51.08
		ViT Encoder Block	41.70	27.31	61.30	52.20
	MSVD	LSTM	54.80	33.90	73.00	96.30
		ViT Encoder Block	54.60	35.80	72.90	97.00

**Table 9:** Quantitative results of fixed Backbone network usage metric learning and different encoder under MSR-VTT and MSVD benchmark dataset.

Backbones	Dataset	Encoder	Score			
			B4	M	R	C
SE_ResNet152+ResNeXt-101+I3D	MSR-VTT	LSTM	41.50	28.40	61.80	52.80
		ViT Encoder Block	42.20	28.90	62.16	54.30
	MSVD	LSTM	54.80	35.80	73.30	97.50
		ViT Encoder Block	55.30	36.10	74.20	98.40

**Table 10:** Quantitative results of the metric learning module with fixed Backbone network and encoder architecture under the MSR-VTT and MSVD benchmark dataset.

Backbones	Encoder	Dataset	Metric learning	Score			
				B4	M	R	C
SE_ResNet152+ResNeXt101+I3D	ViT	MSR-VTT	NO	41.70	27.31	61.30	52.20
			YES	42.20	28.90	62.16	54.30
		MSVD	No	54.60	35.80	72.90	97.00
			YES	55.30	36.10	74.20	98.40

## Conclusions and future work

We propose a novel semantic guidance network for video captioning, alleviating existing methods that cannot make good use of the visual representation and long-distance encoding. Our model consists of a scene frame selection module, visual feature and semantic information extraction module, ViT encoder block encoder, and LSTM decoder module. Specifically, the proposed model can make full capture of representation information of each scene and achieve the longer-distance dependencies encode representation. In addition, a non-parametric metric learning module is used to further optimize the description performance of the proposed model. Extensive experiments verify the description effectiveness of the proposed method on two mainstream datasets MSR-VTT and MSVD. Furthermore, the description performance is further verified on the real-world engineering application datasets LSMDC.

In future works, we will focus on the following directions. First, novel attention mechanisms and reinforcement learning methods can be used to further improve the descriptive performance of the proposed model. Second, the domain adaptation strategy seems a better choice for video captioning.

## Data Availability

Because the video data set occupies a large amount of storage, the datasets used and/or analyzed during the current study are available from the corresponding author upon reasonable request.
